# Tetrandrine induces autophagy and differentiation by activating ROS and Notch1 signaling in leukemia cells

**DOI:** 10.18632/oncotarget.3505

**Published:** 2015-03-10

**Authors:** Ting Liu, Qiuxu Men, Guixian Wu, Chunrong Yu, Zan Huang, Xin Liu, Wenhua Li

**Affiliations:** ^1^ College of Life Sciences, Wuhan University, Wuhan, P. R. China; ^2^ Ministry of Education Laboratory of Combinatorial Biosynthesis and Drug Discovery, College of Pharmacy, Wuhan University, Wuhan, P. R. China

**Keywords:** tetrandrine, leukemia cells, autophagy, differentiation

## Abstract

All-trans retinoic acid (ATRA) is a differentiating agent for the treatment of acute promyelocytic leukemia (APL). However, the therapeutic efficacy of ATRA has limitations. Tetrandrine is a traditional Chinese medicinal herb extract with antitumor effects. In this study, we investigated the effects of tetrandrine on human PML-RARα-positive acute promyelocytic leukemia cells. Tetrandrine inhibited tumors in vivo. It induced autophagy and differentiation by triggering ROS generation and activating Notch1 signaling. Tetrandrine induced autophagy and differentiation in M5 type patient primary leukemia cells. The *in vivo* results indicated that low concentrations of tetrandrine inhibited leukemia cells proliferation and induced autophagy and then facilitated their differentiation, by activating ROS and Notch1 signaling. We suggest that tetrandrine is a potential agent for the treatment of APL by inducing differentiation of leukemia cells.

## INTRODUCTION

Acute myeloid leukemia (AML) is a heterogeneous clonal disorder of hemopoietic progenitor cells. The clonal disorder is resulting from the accumulation of clonal myeloid progenitor cells that are unable to differentiate normally. AML is the most common malignant myeloid disorder in adults [1]. Acute promyelocytic leukemia (APL) is a distinct subtype of AML that is characterized by a specific chromosome translocation, which results in the fusion of the promyelocytic leukemia (PML) gene and the retinoic acid receptor α gene (RARα) [2]. The aberrant oncogenic protein (PML-RARα) blocks further myeloid differentiation at the promyelocyte stage [3]. Currently, NB4 cell line is a good model to study APL, as it is a human PML–RAR α-positive APL cell line.

Differentiation therapy focuses on forcing cancer cells to restore their differentiation capacity and achieve terminal differentiation, which has successfully been applied in APL. Initially, APL became the first model of a malignant disease responding to a differentiation agent [4, 5]. Differentiation agents are involved in the reprogramming of cancer cells, which results in repressing their proliferation and inducing the terminal maturation of cells, even inducing apoptosis and, ultimately, cell death [6-8]. Differentiation therapy is less toxic than classical chemotherapy, which not only kills cancer cells but also has side effects on normal cells. Clinically, all-trans retinoic acid (ATRA) alone or in combination with arsenic trioxide (ATO) treatment targets the RARα and induces terminal differentiation of APL blasts [9-12]. Although the rate of remission for APL patients after ATRA treatment is high, the therapeutic efficacy of ATRA still has some challenges, including liver toxicity, secondary resistance and some cases of ATRA-resistant APL patients [13-15]. The gradually revealed limitations of ATRA treatment for APL patients indicates that it is necessary to find more useful therapeutic agents that have the capacity to increase the level of ATRA sensitivity of leukemic cells or have the ability to “cure” APL on their own [16].

Autophagy is a ubiquitous and dynamic cellular process that involves the rearrangement of subcellular components and organelles in cytoplasm for delivery to the lysosome or vacuoles where the sequestered cargo, such as proteins and lipids, are degraded and recycled [17, 18]. Autophagy is a double-edged sword in the modulation of cancer; experimental evidence supports a role for autophagy in both cancer development and suppression [19]. Increasing evidence proved that activation of autophagy by multiple signaling pathways is a potential therapeutic strategy for cancer [20-22]. It been demonstrated that autophagy regulates myeloid cell differentiation by p62/SQSTM1-mediated degradation of the PML-RARα oncoprotein [23, 24]. Specific roles for autophagy have also been identified in lymphocytes, plasma and monocyte–macrophage cell differentiation [25-28]. Therefore, enhancing autophagy may be a promising therapeutic treatment for facilitating cellular differentiation in resistant APL.

Tetrandrine, a bisbenzylisoquinoline alkaloid, is a key ingredient isolated from the roots of broadly used traditional Chinese medicinal herb Stephaniae tetrandrae [29-31], which exhibits potent antitumor effects. We previously reported that high concentrations of tetrandrine effectively induce apoptosis, and low doses of tetrandrine induce cellular autophagy in liver cancer cells and show good synergistic antitumor effects in combination with other chemotherapeutic agents [32-34]. Interestingly, we also found that tetrandrine is a potent agonist for cell autophagy in many types of cancer cells and even exhibits a much stronger activity than rapamycin for inducing autophagy [35].

In the present study, we investigated the efficacy of tetrandrine on NB4 cells *in vivo* and *in vitro*. The results revealed that tetrandrine has the capacity to induce differentiation accompanied by autophagy and inhibit proliferation in NB4 cells. Reactive oxygen species (ROS) accumulation and Notch1 signaling activation were involved in the tetrandrine-induced autophagy and differentiation. Moreover, tetrandrine also induced autophagy and differentiation in M5 type patient primary leukemia cells. These findings provide a novel chemotherapeutic strategy for APL, even in AML patients, with the development of tetrandrine as a differentiation-inducing agent.

## RESULTS

### Tetrandrine induces NB4 cell autophagy and differentiation and represses tumor growth *in vivo*

**Fig.1:**
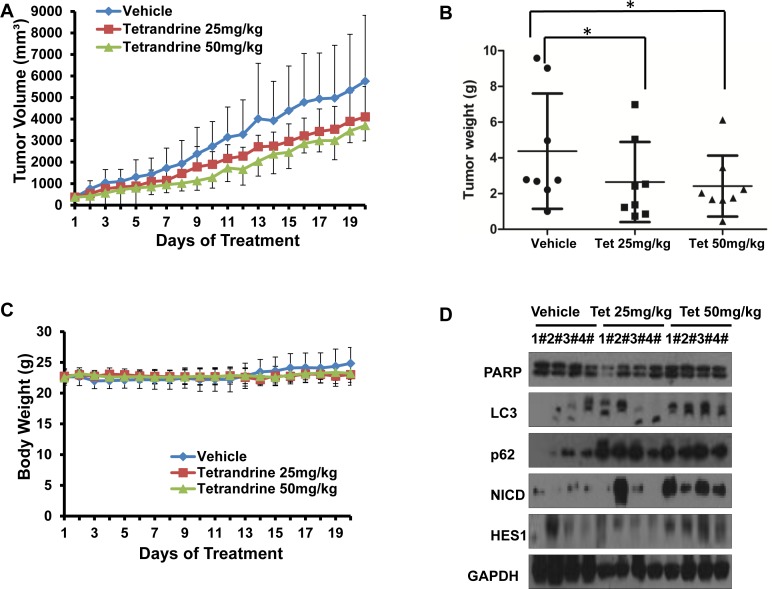

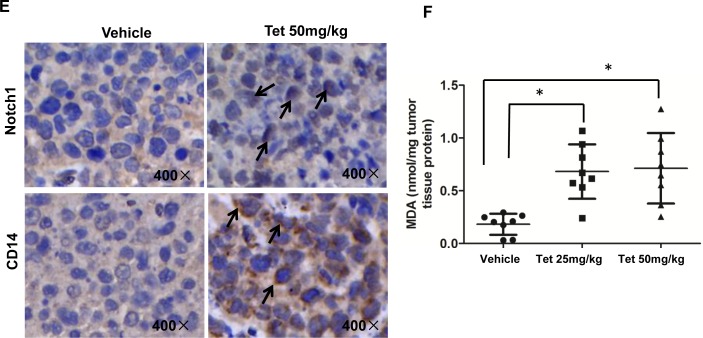
Tetrandrine induces NB4 cell autophagy and differentiation and represses tumor growth *in vivo* NB4 cells were inoculated into mice to establish a tumor model, as indicated in the Materials and Methods. Mice bearing tumors were randomly placed into three groups (8 mice /group) and were treated daily with vehicle or tetrandrine (25 mg/kg or 50 mg/kg) for 20 days. Animal weight and tumor volume was measured daily. (A) Mean tumor volumes after treatment. Values represent the means ± SD.*p<0.05. (B) Tumor weight after 20 days of treatment are presented in a scatter plot; the bars represent the ±SD. *p<0.05. (C) The mean values of the mouse body weights. (D) Western blot analysis of tissue lysates isolated from 25 (Tet 25 mg/kg) or 50 mg/kg (Tet 50 mg/kg) tetrandrine-treated or vehicle-treated NB4 cell xenografts. 4 mice in each group have been defined as “1#” to “4#”, respectively. (E) Notch1 (both full length and NICD proteins) levels and CD14 were evaluated by immunohistochemistry in tumor tissues derived from the 50 mg/kg tetrandrine-treated and the control mouse models. Magnification: × 400. (F) MDA level of tumor tissue proteins exacted from NB4 tumor xenografts. *p<0.05.

To evaluate the anti-tumor effects of tetrandrine *in vivo*, we established NB4 subcutaneous tumor xenograft models with athymic nude mice. Nude mice bearing NB4 tumors (approximate volume 50 mm^3^) were randomly divided into three groups (8 mice/group) and were intragastrically treated with vehicle or tetrandrine (25 mg/kg or 50 mg/kg) once daily for 20 days. Mouse body weight and tumor size were measured daily. As shown as Fig. 1A, tetrandrine inhibited tumor growth. Consistent with the tumor volume results, tetrandrine treatment also led to a slowed the increase in tumor weight (Fig. 1B). Notably, we found no additional weight loss or other signs of toxicity, even in mice treated with 50 mg/kg tetrandrine for 3 weeks (Fig. 1C). Additionally, Western blot analysis of tumor tissue samples found that tetrandrine increased LC3-II protein levels and activated the Notch1 signaling pathway (Fig. 1D). Moreover, tetrandrine also upregulated CD14 expression on the NB4 cell surface *in vivo* (Fig. 1E). Additionally, the level of the lipid peroxidation product MDA, used as a presumptive measure of ROS, was increased in tumor tissues upon tetrandrine treatment (Fig. 1F). These observations suggest that tetrandrine exhibited good anti-tumor activity *in vivo*, and the potential mechanism was associated with the induction of tumor cell autophagy and differentiation by triggering ROS generation and activation of Notch1 signaling.

### Tetrandrine-induced autophagy and differentiation in M5 type patient primary leukemia cells

**Fig.2:**
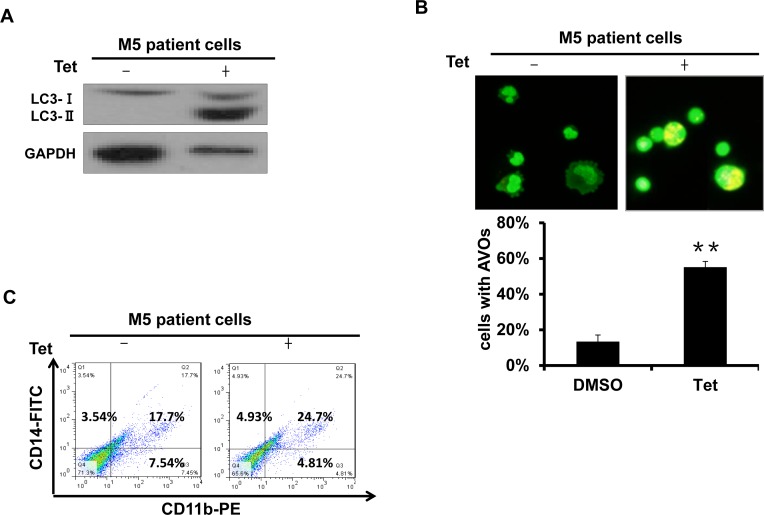
Tetrandrine induced autophagy and differentiation in M5 type patient primary leukemia cells M5 leukemia is one subtype of acute myeloid leukemia (AML). (A) Western blot analysis of LC3 protein levels. M5 type patient cells were treated with 2 μM tetrandrine (Tet) for 24 hours. (B) Acridine orange staining assay analysis of autophagy in M5 type patient cells. Cells were treated with 2 μM tetrandrine (Tet) for 24 hours. Error bars represent the mean ±SD. **p <0.01. (C) Flow cytometry analysis of CD11b and CD14 expression. M5 type patient cells were treated with 2 μM tetrandrine (Tet) for 4 days.

M5 leukemia, or acute monocytic leukemia, is one of the most common subtypes of AML. To assess the effects of tetrandrine on human primary leukemia cells, we treated primary leukemia cells obtained from M5 type patients who had not previously received chemotherapy. The results showed that tetrandrine treatment dramatically promoted LC3 protein expression as well as an increased accumulation of acidic autophagolysosome vacuoles (Fig. 2A and B). In addition, tetrandrine treatment also facilitated the expression of CD11b and CD14 on the surface of M5 type patient cells (Fig. 2C). These results revealed that tetrandrine exhibited considerable effects on the differentiation of M5 type patient primary leukemia cells.

### A 2 μM concentration of tetrandrine inhibits the proliferation of the NB4 acute promyelocytic leukemia cell line

**Fig.3:**
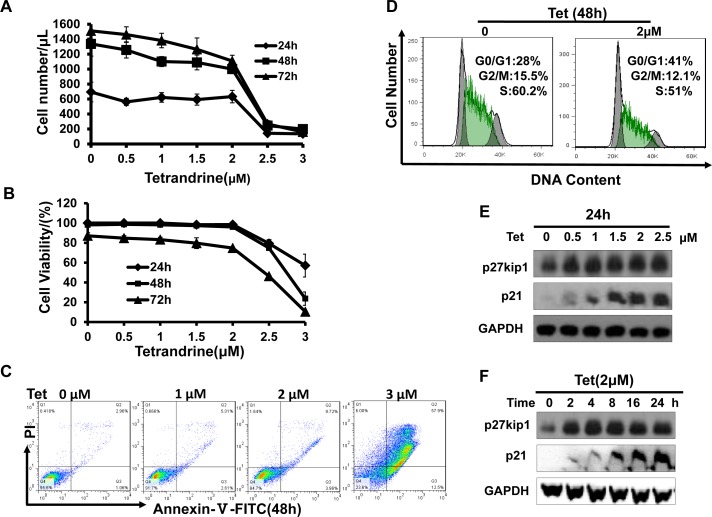
Tetrandrine (2 μM) inhibits acute promyelocytic leukemia NB4 cell proliferation Data are representative of values from at least three independent experiments. (A) NB4 cells were treated with 0 (DMSO), 0.5, 1, 1.5, 2, 2.5, or 3 μM tetrandrine for 24, 48 and 72 hours. The results show anti-proliferation effects (B) but minimal effects on cell viability. Data are represented as mean ± SD. (C) NB4 cells were treated with 0 (DMSO), 1, 2, or 3 μM tetrandrine (Tet) for 48 hours. Apoptosis was determined by annexin V-FITC/PI staining for 15 min at room temperature. (D) Flow cytometry analysis of cell cycle in NB4 cells after 2 μM tetrandrine (Tet) treatment for 48 h. (E) Western blot analysis of the cell cycle-related proteins p27kip1 and p21 in NB4 cells showed dose-dependent and (F) time-dependent increases after tetrandrine (Tet) treatment.

We have previously reported that tetrandrine exhibits potent antitumor effects in solid cancer. To determine the effects of tetrandrine on acute promyelocytic leukemia, NB4 cells were treated with 0 (DMSO), 0.5, 1, 1.5, 2, 2.5, or 3 μM tetrandrine for 24, 48 or 72 hours. The results showed that tetrandrine inhibited cell proliferation at 0.5-2 μM (Fig. 3A). To exclude the possibility that cell death reduced the number of cells, cell viability was assayed and the result showed that tetrandrine hardly kill NB4 cells at concentrations of 0-2 μM (Fig. 3B). Thus, we concluded that tetrandrine primarily inhibited NB4 cell proliferation at a 2 μM concentration. However, 3 μM tetrandrine significantly induced apoptosis in NB4 cells (Fig. 3C). Further cell cycle analysis indicated that 2 μM tetrandrine treatment for 48 hours resulted in a G0/G1 phase arrest of NB4 cells (Fig. 3D). Western blots demonstrated that tetrandrine dramatically promoted the expression of the cyclin-dependent-kinase (CDK) inhibitor p27kip1 and the p21 protein (Fig. 3E and F) but did not affect cyclinD, cyclinE and CDKs protein levels (data not shown). Therefore, we speculated that tetrandrine inhibits NB4 cell proliferation by regulating p27kip1 and p21 expression to cause cell cycle arrest at the G0/G1 phase.

### A 2 μM concentration of tetrandrine induces NB4 cell autophagy

**Fig.4:**
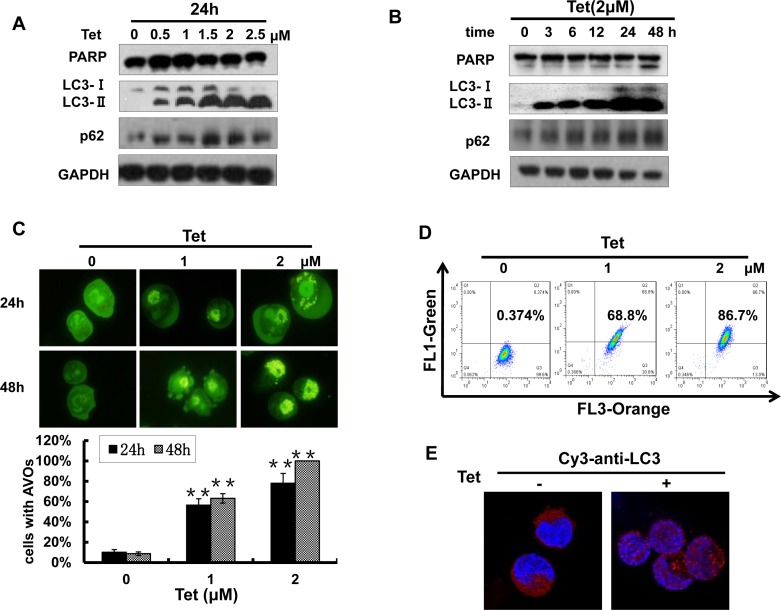
Tetrandrine (2 μM) induces NB4 cell autophagy (A) Western blot analysis of the autophagy-related proteins LC3, p62 and the apoptosis-related protein PARP levels in NB4 cells after treatment with a tetrandrine (Tet) concentration gradient (0-2.5 μM) and (B) different time intervals (0-48 h). (C) Tetrandrine (Tet) treatment induces autophagy in NB4 cells as analyzed by acridine orange staining assays viewed under a fluorescent microscope and (D) measured by flow cytometry. Data are represented as mean ± SD. (E) Immunofluorescence staining for LC3 (Cy3-anti-LC3 antibody) to analyze autophagy by confocal microscopy after 2 μM tetrandrine (Tet) treatment for 24h.

Many chemotherapeutic agents have the capability to induce cancer cell autophagy, which plays an important role in cancer treatment. Next, we determined whether tetrandrine treatment was able to induce NB4 cell autophagy. As shown in Fig. 4A and B, tetrandrine markedly increased the levels of LC3-II and p62, which are established markers of cell autophagy, in a dose- and time-dependent manner. However, poly (ADP)-ribose polymerase (PARP), the marker for apoptosis, was not increased with 2 μM tetrandrine treatment. To further confirm this observation, we stained NB4 cells with acridine orange and detected the formation of numerous acidic autophagolysosome vacuoles (AVOs) with multiple test methods. The data showed that 2 μM tetrandrine resulted in a considerable accumulation of intracellular autophagic vacuoles (Fig. 4C, D and E). The above results findings indicate that 2 μM tetrandrine led to NB4 cell autophagy.

### Tetrandrine facilitates NB4 cell differentiation

**Fig.5:**
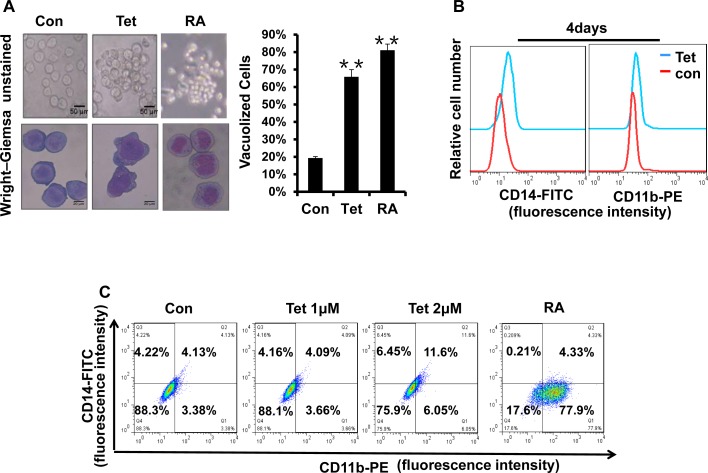

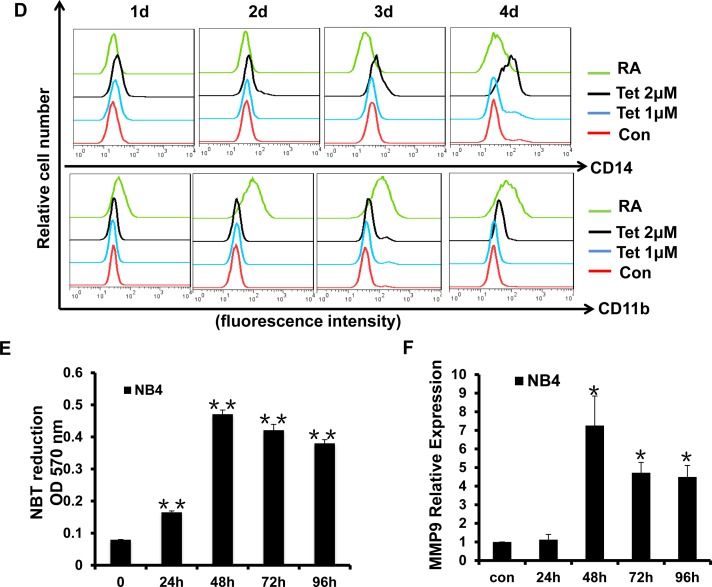
Tetrandrine facilitates NB4 cell differentiation Oxidant dimethylsulfoxide (DMSO) was used as a negative control (Con), and All-trans retinoic acid (RA) treatment was used as a positive control. (A) Cell morphology was observed under a microscope with or without Wright–Giemsa staining after 4 days of 2 μM tetrandrine (Tet) treatment. Data are represented as mean ± SD. **p <0.01. (B) CD11b and CD14 antigens expression was measured alone (C) or co-measured by flow cytometry after 4 days of tetrandrine (Tet) treatment. (D) The respective cell surface expression of the CD11b and CD14 antigens, on NB4 cells after tetrandrine (Tet) treatment at the indicated concentrations and times was measured by flow cytometry. (E) NBT reduction assay analysis of differentiation. NB4 cells treated with 2 μM tetrandrine (Tet) for 24h-96h. Data are represented as mean ± SD. **p <0.01. (F) The relative levels of MMP9 expression were measured by RT-PCR after 2 μM tetrandrine (Tet) treatment for 24h-96h. Data are represented as mean ±SD. *p <0.05.

The strategy of inducing the differentiation of leukemia cells is significant for APL clinical treatment. To examine whether tetrandrine could stimulate the differentiation of NB4 cells, we first evaluated cellular maturation by observing the cellular morphology with Wright-Giemsa staining. As shown in Fig. 5A, NB4 cells treated with tetrandrine had the typical characteristic morphology of differentiated cells, such as an irregular nucleus, the presence of vacuoles and a large nuclear/cytoplasm ratio. Next, we further predicted NB4 cell differentiation by analyzing the expression of the cell surface differentiation-related antigens, CD14 and CD11b. The results revealed that 2 μM tetrandrine enhanced CD14 and CD11b expression on the NB4 cell surface, either alone or in combination, as shown by immunofluorescence labeling of these two proteins. In contrast, 1 μM tetrandrine did not significantly induce NB4 cell differentiation (Fig. 5B, C and D). The NBT reduction assay, which is a classic experiment to detect cellular differentiation, also found that NBT reduction activity increased remarkably with tetrandrine treatment and reached a peak value at 48 hours (Fig. 5E). MMP9 levels can be used as an additional NB4 cell differentiation marker. Here, we also observed that tetrandrine treatment promoted a significant increase of MMP9 mRNA levels (Fig. 5F). Thus, these results suggest that 2 μM tetrandrine promoted NB4 cell differentiation.

### Early stage autophagy is related to tetrandrine-induced differentiation

**Fig.6:**
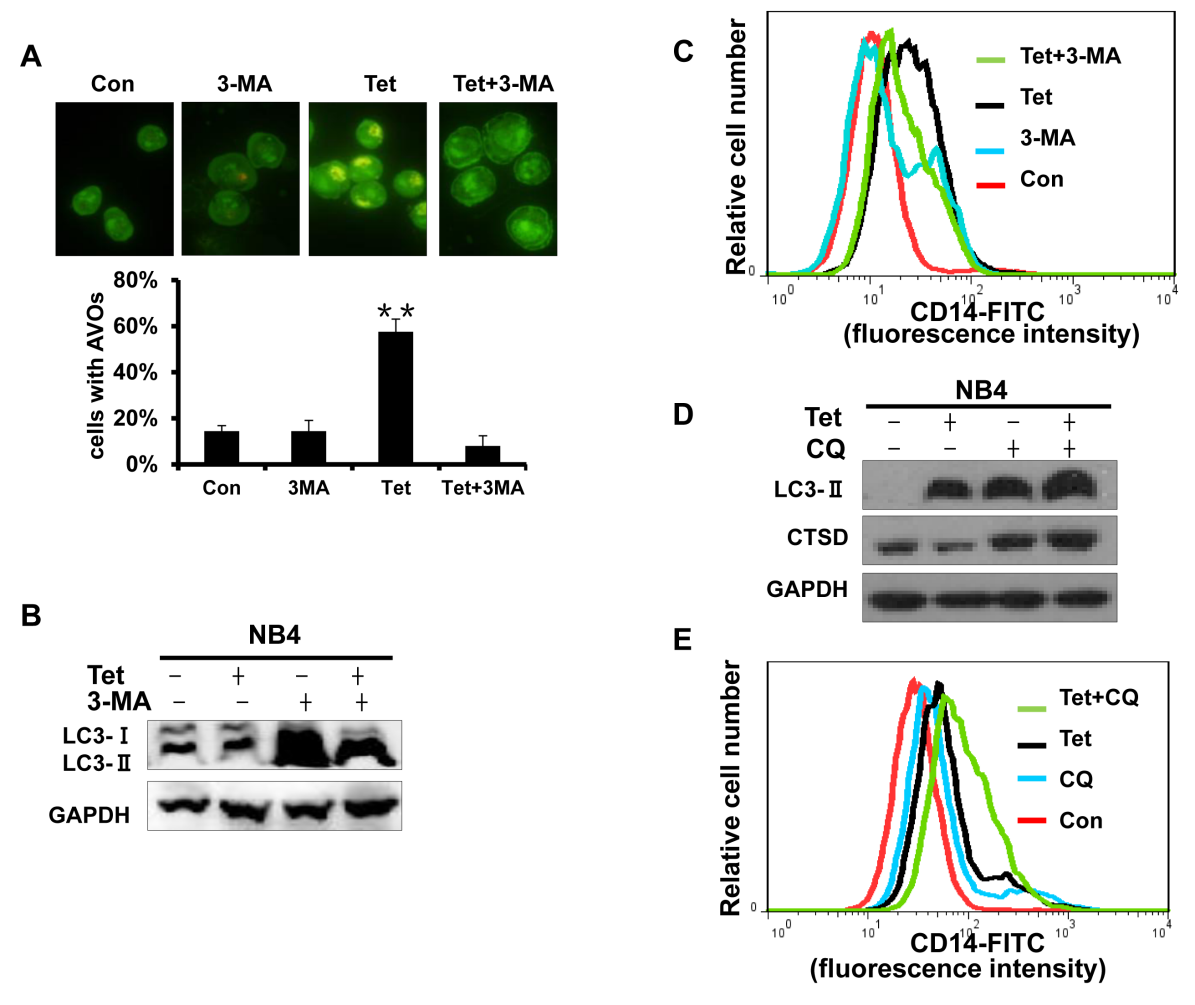
Early stage autophagy is related to tetrandrine-induced differentiation (A) Acridine orange staining assay analysis of autophagy. NB4 cells treated with Oxidant dimethylsulfoxide (Con), 2 μM tetrandrine (Tet), 1.5 mM 3-methyladenine (3-MA), and 2 μM tetrandrine (Tet) after a 1-hour pretreatment with 1.5 mM 3-MA (Tet+ 3-MA) for 10 hours. Error bars represent the mean ±SD. **p <0.01. (B) Western blot analysis of tetrandrine (Tet) induced LC3-II protein levels in the presence or absence of 1.5 mM 3-MA pretreatment for 10 hours. (C) CD14 was detected by flow cytometry of NB4 cells pretreated with 3-MA and incubated with tetrandrine (2 μM) for 4 days. (D) Western blot analysis of LC3-II and CTSD protein levels. Cells were treated with DMSO (Con), 2 μM tetrandrine (Tet), 15 mM chloroquine (CQ), and 2 μM tetrandrine (Tet) after a 1-hour pretreatment with 15 mM chloroquine (Tet+ CQ) for 24 hours. (E) CD14 was measured by flow cytometry of NB4 cells that were pretreated with 15 mM CQ and then treated with tetrandrine (2 μM) for 4 days.

Recent reports show that autophagy is a potentially important regulator of differentiation in anti-leukemic strategies. Therefore, we next investigated whether tetrandrine-induced differentiation is related to autophagy. 3-methyladenine (3-MA), a common specific inhibitor of early stage autophagy through the blockade of autophagosome maturation, was used to block tetrandrine-induced autophagy. The results showed that 3-MA not only significantly prevented the formation of the AVO fluorescent orange puncta and the LC3-II protein level (Fig. 6A and B) but also inhibited tetrandrine-induced differentiation (Fig. 6C). To examine the relationship of late-stage autophagy and cell differentiation, we treated NB4 cells with the lysosome inhibitor chloroquine (CQ) to inhibit late autophagy/lysosomal protein degradation and break the autophagic flux. As shown as Fig. 6D and E, while CQ blocked late-stage autophagic flux, it showed a synergistic induction of differentiation in combination with tetrandrine. These findings suggest that tetrandrine-induced NB4 cell differentiation is associated with early stage but not late-stage autophagy.

### The activation of Notch1 signaling is involved in tetrandrine-induced NB4 cell autophagy and differentiation

**Fig.7:**
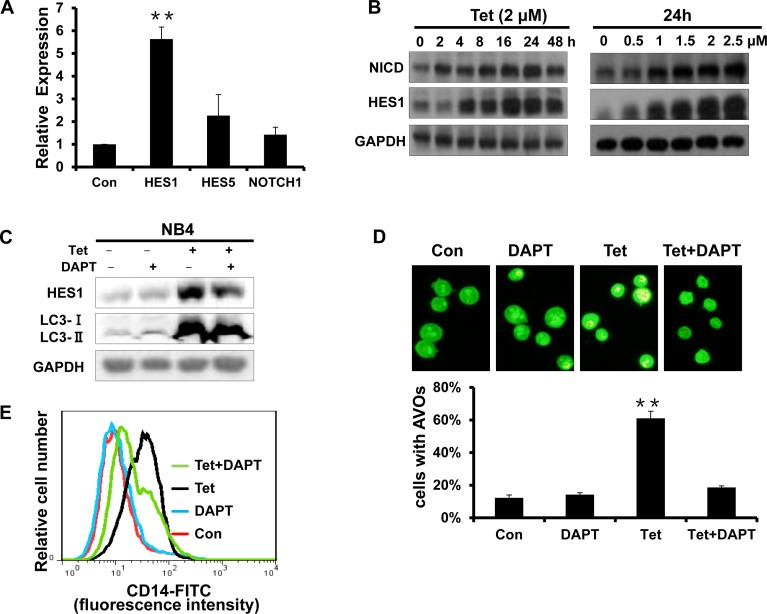
The activation of Notch1 signaling is involved in tetrandrine-induced NB4 cell autophagy and differentiation (A) RT-PCR analysis of the relative mRNA expression levels of HES1, HES5 and Notch1. NB4 cells were exposed to 2μM tetrandrine for 24 hours. Oxidant dimethylsulfoxide (DMSO) was used as a negative control (Con). Error bars represent the mean ±SD. **p <0.01. (B) Western blot analysis of NICD and HES1 protein levels after tetrandrine (Tet) treatment at the indicated doses and time intervals. (C) Western blot analysis of HES1 and LC3 levels. NB4 cells were treated with DMSO (Con), 2 μM tetrandrine (Tet), 2 mM DAPT, and 2 μM tetrandrine (Tet) after a 1-hour pretreatment with 2 mM DAPT (Tet+ DAPT) for 24 hours. (D) Acridine orange staining assay analysis of autophagy. NB4 cells treated with 2 μM tetrandrine (Tet) after a 1-hour pretreatment with 2 mM DAPT. Error bars represent the mean ±SD. **p <0.01. (E) NB4 cells were 1-hour pretreated with DAPT and incubated with 2 μM tetrandrine (Tet) for 4 days prior to CD14 detection by flow cytometry.

Notch is a critical signaling intermediate that regulates hematopoietic cell differentiation through downstream signal transduction cascades. To determine the role of Notch1 signaling activity in tetrandrine-induced cell autophagy and differentiation, we evaluated the mRNA levels of HES1, HES5 and Notch1. As shown in Fig. 7A, tetrandrine treatment dramatically up-regulated HES1 mRNA levels. Western blots also showed that tetrandrine treatment increased the levels of the NICD and HES1 proteins (Fig. 7B), which suggests that tetrandrine activates Notch1 signaling. Interestingly, pretreatment with DAPT, an established Notch signaling inhibitor, partially decreased the tetrandrine-induced levels of the LC3-II protein and the numbers of AVO fluorescent orange puncta (Fig. 7C and D) and down-regulated the levels of CD14 expression on the surface of NB4 cells (Fig. 7E). Therefore, these results indicated that Notch1 signaling is most likely involved in tetrandrine-induced NB4 cell autophagy and differentiation.

### Intracellular ROS generation is an early event in tetrandrine-induced cellular autophagy and differentiation

**Fig.8:**
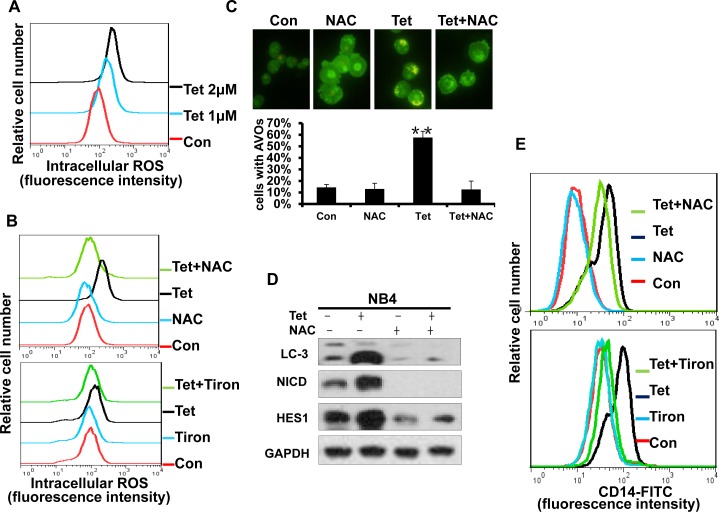
Intracellular ROS generation is an early event of tetrandrine-induced cell autophagy and differentiation (A) Effects of tetrandrine (1, 2 μM) on intracellular ROS levels after 24 hours treatment. (B) NB4 cells were treated with DMSO (Con), 2 μM tetrandrine (Tet), 10 mM N-acetyl cysteine (NAC) or 0.2 mM Tiron, and 2 μM tetrandrine (Tet) after a 1-hour pretreatment with 10 mM NAC or 0.2 mM Tiron (Tet+ NAC or Tiron) for 24 hours. Intracellular ROS levels were measured by flow cytometry and (C) autophagy was detected with acridine orange staining assays. Error bars represent the mean ±SD. **p <0.01. (D) Western blot analysis of LC3, NICD and HES1 levels in cells after 1-hour pretreated with NAC for 24 hours. (E) Flow cytometry analysis of CD14 antigens expression on the surface of NB4 cells after 4days treatment.

Some chemotherapeutic agents can induce ROS generation in certain types of cancer cells, and excess ROS will trigger subsequent physiological cell signaling that regulates cell proliferation, survival and differentiation. Therefore, we next investigated whether ROS activation played an essential role in tetrandrine-induced NB4 cell autophagy and differentiation. Using H_2_DCFDA-based detection by flow cytometry, dose-dependent ROS accumulation was observed after tetrandrine treatment of NB4 cells (Fig. 8A). The free radical scavenger NAC and Tiron markedly abrogated tetrandrine-induced ROS generation (Fig. 8B). Importantly, pretreatment with NAC and Tiron markedly blocked tetrandrine-induced cell autophagy by decreasing AVO fluorescent orange puncta and the levels of the LC3-II protein and prevented cell differentiation by down-regulating the expression of CD14 on the surface that tetrandrine-induced NB4 cells (Fig. 8C, D and E). Moreover, Western blot results also showed that NAC notably recovered the tetrandrine-induced increase in NICD and HES1 protein levels (Fig. 8D). Taken together, these results demonstrated that activation of intracellular ROS is essential for tetrandrine-induced cell autophagy and differentiation and that ROS act upstream of Notch1 signaling.

## DISCUSSION

All-trans-retinoic acid (ATRA) is currently the most efficient agent used in differentiation therapy of acute myeloid leukemia (AML). Although higher clinical efficiency in APL patients is achieved by a combination of ATRA with arsenic trioxide (ATO) [12, 36, 37], the development of chemotherapeutic differentiation therapies is still limited. Hence, developing specific agents that can trigger differentiation of leukemic cells along normal hematopoietic lineages has become necessary. Tetrandrine was suggested to be an autophagy agonist for solid tumors in our previous studies. Here, we demonstrated that tetrandrine had notable effects on NB4 tumor growth repression, including the induction of cell cycle arrest, autophagy, differentiation, ROS accumulation, and Notch1 signaling activation, not only *in vitro* but also *in vivo*. Some other reports indicated that the ROS scavenger NAC can inhibits mTOR [38], but it proved in our system that accumulation of intracellular reactive oxygen species (ROS) involved in tetrandrine induced autophagy and NAC can block this autophagy induction without inducing significant autophagy itself, and whether NAC can activate autophagy by inhibiting mTOR needs futher exploration. As tetrandrine comes from natural plant product, it has multiple bioactivities such as anti-inflammatory, anti-allergic, anti-fibrogenic, and antitumor activities. Anticancer activity is most hot area among the masses of pharmacological effects displayed by tetrandrine. It may have multi-targets of treating cancer and the cells are undergoing various forms of dynamic process after drug treatment. Differentiation is one of the main points in tetrandrine treated NB4 cells, as well as proliferation inhibition and autophagy induction, but other targets may still exist and will be functional. Tetrandrine displays these effects through selective modulation of multiple signaling pathways. Anti-tumor is the sum of a variety of effects. Since most tumorigenesis involved complicated process, targeting drug may not enough to approach effective therapeutic effect, multiple bioactivities attributed to drugs such as tetrandrine actually make tetrandrine become a potent candidate in therapeutics. Therefore, our findings indicated that in addition to solid cancer, tetrandrine also exhibits potent antitumor effects on acute promyelocytic leukemia and may provide a novel chemotherapeutic strategy for APL patients.

Because APL pathogenesis includes blocking the myeloid differentiation program and enhancing the self-renewal of leukemic cells, targeting differentiation is an effective therapy for APL. To evaluate the potential of tetrandrine to induce NB4 leukemia cell differentiation, we examined CD14 and CD11b expression on the cell surface. Although a slight change of CD11b was observed after tetrandrine treatment, the membrane antigen density of CD14 was markedly increased, which suggested that NB4 cells differentiated into mature monocytes and macrophages. In contrast, ATRA effectively facilitated the differentiation of APL cells into mature granulocytes, as assessed by CD11b surface expression. Previous reports showed that ATRA induced APL cell differentiation by activation of RARα and induced the degradation of PML-RARα by changing epigenetic modifiers from co-repressor complexes to co-activator complexes on target genes. Here, we demonstrated that tetrandrine promoted NB4 cell differentiation primarily through triggering ROS and Notch1 signaling pathways. Therefore, we hypothesized that tetrandrine had different molecular mechanisms of inducing leukemia cell differentiation compared with ATRA. More interestingly, tetrandrine-induced leukemia cell differentiation is very likely autophagy-dependent.

Autophagy is one of several proteolytic mechanisms and is an efficient way to promote the degradation of the redundant abnormal oncogenic fusion protein that promotes the further differentiation of abnormal cells. The elimination of the special PML-RARα oncogenic fusion protein is critical to obtain complete and sustained APL remission [20, 39]. P62/SQSTM1 is an ubiquitin-binding adaptor protein that binds ubiquitin and regulates signaling cascades through ubiquitination. P62 connects the ubiquitin-proteasome and autophagy-lysosome pathways to mediate degradation of intracellular proteins. In our study, after tetrandrine-induced autophagy was blocked by 3-MA (a common specific inhibitor of autophagy at early stage) pretreatment in the autophagosome formation stage, tetrandrine-induced differentiation also decreased, which indicated that tetrandrine-induced autophagy contributes to the differentiation of leukemia cells. However, CQ treatment to inhibit autophagy-lysosome protein degradation showed that tetrandrine and CQ were synergistic for inducing differentiation. This may be explained because the CQ autophagy inhibitor increased the accumulation of some proteins that are dependent on the inhibition of autophagosome maturation and degradation, which benefits cell differentiation by potentiating drug-induced growth arrest and the differentiation of leukemic cells [4, 40]. Thus, it is possible that autophagy is involved in the breakdown of aggregates of oncogenic fusion proteins, such as PML–RAR α, and also takes part in other pathways that have effects on cell differentiation.

Notch signaling is a highly evolutionarily conserved pathway that is involved in stem cell maintenance and developmental processes by regulating cell fate decisions [41]. Although Notch signaling has been shown to play both oncogenic and tumor suppressor roles in cancer, depending on the cell type, increasing evidence has demonstrated the importance of Notch1 signaling in hematopoietic development and leukemia therapy [42], especially in AML and T-cell acute lymphoblastic leukemia (T-ALL) [43, 44]. Moreover, Notch1 agonists have been reported as a potential therapeutic approach in AML [45, 46]. In our studies, we found that tetrandrine induced NB4 differentiation by activating Notch1 signaling, which confirmed that Notch1 signaling may be an APL suppressor pathway. Though the detailed molecular mechanisms need to be further explored, the present results are sufficient to suggest that tetrandrine or other Notch signaling inducers possess good therapeutic prospects for leukemia patients in clinical treatment.

In conclusion, following our previous report on the role of tetrandrine in human hepatocellular carcinoma, we show here that tetrandrine is a promising chemotherapeutic agent for acute promyelocytic leukemia. Tetrandrine can inhibit NB4 cell growth and induces autophagy-associated differentiation both *in vitro* and *in vivo*. The potential molecular mechanisms of tetrandrine involve ROS accumulation and Notch1 signaling activation, which acted as an essential initial signal to facilitate tetrandrine-induced NB4 cell autophagy and differentiation. Therefore, our data broaden the application of tetrandrine in clinical therapies and provide the rationale for the therapeutic regimens for leukemia patients as well.

## MATERIALS AND METHODS

### Chemical reagents and antibodies

Tetrandrine was purchased from Shanghai Ronghe Medical, Inc. (Shanghai, China) and was stored at −80°C as a powder and dissolved in dimethyl sulfoxide before use. 3-Methyladenine and N-acetyl-L-cysteine were purchased from Sigma (St. Louis, MO). DCFH-DA was obtained from Invitrogen (Carlsbad, CA). DAPT was obtained from Selleck (Shanghai, China). Acridine orange, NBT, the GAPDH antibody and HRP-conjugated secondary antibodies (goat anti-rabbit and goat anti-mouse) were purchased from Beyotime (Nantong, China). The antibody against microtubule-associated protein 1 light chain 3 (LC3) was purchased from Sigma (St. Louis, MO). CD14-FITC and CD11b-PE were obtained from BD Biosciences. Other antibodies were obtained from the following sources: p27kip1, PARP, p62, HES1, Notch1 were from Cell Signaling Technologies (Beverly, MA); CD14, cathepsin D (CTSD), and p21 were acquired from Proteintech Group Inc. (Chicago, IL).

### Cell line and cell culture

NB4 cells were kindly provided by Dr. Zan Huang (Wuhan University). Cells were cultured at 37 °C in a humidified atmosphere of 95% air and 5% CO_2_ in complete RPMI 1640 medium (Gibco BRL, Grand Island, NY, USA), which was supplemented with 10% fetal bovine serum (FBS, Hyclone), 1% penicillin and 1% streptomycin. Cell culture dishes and plates were obtained from Wuxi NEST Biotechnology (Co., Ltd).

### Cell proliferation and cell viability analysis

Cell proliferation was measured by counting the total number of cells. Cell viability was observed by the trypan blue dye-exclusion assay. Cells were plated on 96-well plates and incubated with various concentrations of tetrandrine for the indicated times. A 10μl aliquot of cell suspension was incubated with 10μl 0.4% trypan blue solution for 5 minutes at room temperature. Viable and nonviable cells based on absence and presence of intracellular trypan blue dye, respectively. Percentages were counted by hemacytometer.

### Cell apoptosis assay

For the apoptosis assay, cells were washed with PBS, resuspended in binding buffer from BD Biosciences and stained with Annexin V-FITC and propidium iodide (PI) for 15 min. Annexin V fluorescence was measured with a flow cytometer, and the membrane integrity of the cells was simultaneously assessed by the PI exclusion method.

### Cell cycle analysis

The cell cycle was analyzed by flow cytometry (Guava Technologies, Inc.). After treatment, cells were harvested, washed with PBS and fixed with 70% ethanol overnight at 4 °C. The fixed cells were centrifuged at 800 × g at 25 °C for 5 min, the supernatant was removed, and the cells were washed with PBS. Then, the cells were stained with 4 μl of 10 mg/ml propidium iodide (PI) and 10 μl of 1 mg/ml RNase in 400 μl PBS, followed by incubation in the dark for 30 min prior to measurement of the stained cells on a flow cytometer (Beckman, Indianapolis, CA, USA). The results were analyzed using FlowJo software (Tree Star, San Carlos, USA).

### Western blot analysis

The cells were harvested and lysed in 1% SDS on ice. Then, the cell lysates were heated at 95 °C for 15-20 minutes and then centrifuged at 12,000 x g for 10 minutes. The supernatant was collected, and the protein concentration was determined by the Pierce BCA Protein Assay Kit (Thermo Scientific). Equivalent amounts of protein (20 μg) from each sample were loaded and run on SDS-PAGE gels (Amresco) and transferred to PVDF membranes (Millipore). After blocking the membranes with 5% non-fat milk (Bio-Rad Laboratories, Inc.) in Tris-buffered saline with 0.1% Tween-20 (TBST) at room temperature for 1 hour, the membranes were incubated with specific primary antibodies at 4 °C overnight, washed with TBST three times (10 minutes per wash), and incubated with HRP-conjugated secondary antibodies for 1 hour at room temperature. After washing with TBST, the immunoblots were visualized by chemiluminescence using a HRP substrate (Millipore). GAPDH was probed to ensure equal protein loading.

### Acridine orange staining assays

Acridine orange staining for detecting and quantifying autophagy was measured by fluorescence microscopy and flow cytometry. Autophagy is characterized by the formation of acidic vesicular organelles (AVO), such as autophagosomes and autolysosomes. The intensity of the red fluorescence is proportional to the degree of acidity. Cells were collected and the cell suspension was stained with AO (100 μg/mL) for 15 min. Cells were washed with PBS, resuspended in 0.4 mL PBS and quantified by flow cytometry (Guava technologies). The AO fluorescence was also viewed under a fluorescent microscope (Leica Microsystems GmbH). AVOs were accumulated in acidic spaces and exhibited bright red punctuate fluorescence in the cytoplasm.

### Measurement of ROS accumulation

Intracellular ROS levels were detected by flow cytometry using the DCFH-DA probe (Sigma). Briefly, with the cells were treated with tetrandrine for the indicated time intervals, harvested and washed twice with PBS before being incubated with DCFH-DA (1 μM) in serum-free RPMI 1640 at 37 °C in a 5% CO_2_ incubator for 20 minutes. Next, the cells were washed twice with PBS and analyzed by flow cytometry. The data were processed using the FlowJo software (Tree Star, San Carlos, CA, USA).

### Observation of morphological changes

The cellular morphology of NB4 cells was observed by Wright–Giemsa staining. Cells were fixed with absolute methanol for 30 min, stained with Wright–Giemsa solution (Wako) and observed under a light microscope. ATRA treatment (1 μM) was used as a positive control.

### Differentiation marker analysis

The expression of CD11b and CD14 antigens on the surface of NB4 cells was measured by flow cytometry. Cells were treated for 4 days, and after washing with PBS, 1.0 × 10^5^ cells were labeled with CD14 (CD14-FITC) and CD11b (CD11b-PE) antibodies for 30 min on ice according to the manufacturer's instructions. The cells were then washed twice with ice-cold PBS and finally resuspended in 500 μL PBS for measurement. CD14 and CD11b expression levels were measured using flow cytometry (FACSCalibur, BD Biosciences). FACS data were analyzed with the FlowJo software (Tree Star, San Carlos, USA). ATRA treatment (1 μM) was used as a positive control.

### NBT reduction assay

For the nitroblue tetrazolium (NBT) reduction measurements, cells were treated with tetrandrine for several days in 12-well plates as indicated. After incubation, each cell suspension was mixed with an equal volume of RPMI 1640 containing 1 mg/ml NBT (Sigma) and 20ng/ml TPA for 20 min at 37 °C. The cells were washed in PBS and suspended in 100 μl PBS before evaluation using multimode microplate readers (SpectraMax M5) at 570 nm.

### Quantitative real-time reverse transcription-polymerase chain reaction (RT-PCR)

For quantitative analysis of gene expression, total RNA was isolated using a TRIzol kit (Invitrogen, Carlsbad, CA). Complementary DNA was synthesized using a complementary DNA synthesis kit (Thermo Scientific) according to the manufacturer's instructions. In each reaction, the GAPDH was used as an internal control. The primers used for PCR were as follows: MMP9, 5′-GTCTGCTGAAGTCATCCATCAG-3′ (forward) and 5′-CTTATGTGTAGGAGAGGATAAG-3′ (reverse); HES1, 5′-TGGAAATGACAGTGAAGCACCT-3′ (forward) and 5′-GTTCATGCACTCGCTGAAGC-3′ (reverse); HES5, 5′-GGAAGCCGGTGGTGGAGAAGAT-3′ (forward) and 5′-TCCTGCAGGCACCACGAGTAGC-3′ (reverse); NOTCH1, 5′-GCTGCCTCTTTGATGGCTTCGA-3′ (forward) and 5′-CACATTCGGCACTGTTACAGCC-3′ (reverse) and GAPDH, 5′-TCCAC-CACCCTGTTGCTGTA-3′ (forward) and 5′-ACCACAGTCCATGCCATCAC-3′ (reverse). Relative quantities of mRNA were measured using Applied Biosystems 7500 Fast Real-Time PCR System (PerkinElmer, Torrance, CA) and double-stranded DNA dye SYBR Green PCR core reagents (Roche Life Science, US). Amplification was performed with 40 cycles at 95 ° C for 15 seconds and at 60° C for 30 seconds. Data were analyzed using 7500 system SDS software and the data normalized to GAPDH gene expression.

### Tumor xenograft assay

Animal experiments were conducted according to the guidelines of the Laboratory Animal Center of the Wuhan University College of Life Sciences. Five-week-old male athymic nude mice (BALB/c, nu/nu) were purchased from the Model Animal Research Center (Changsha, China). All experiments were conducted under approved procedures. All qualified mice were injected in the right flank with 1×10^7^ NB4 cells suspended in 0.2 ml of PBS. Tumor growth and the body weight of the mice were monitored every day. Fourteen days later, tumor lumps had reached approximately 50 mm, and tumor-bearing mice were randomly grouped into three experimental categories: vehicle (1% methylcellulose) or one of two concentrations of tetrandrine, 25 or 50 mg/kg body weight tetrandrine. Each group of mice was administered the respective treatment for 3 weeks. Mouse weights and tumor volumes were measured and recorded daily. Tumor volumes were calculated as length*width^2^/2.

### Tissue protein isolation and malondialdehyde (MDA) assay

Mice were sacrificed after 20 days of treatment, and tumor tissues were extracted, washed in PBS and then stored at −80 °C. For the Western blot assay, the tumor tissue was lysed in RIPA buffer on ice and was sonicated to destroy large particles. The cytosol from the tissues was centrifuged at 12,000 x g for 15 min at 4 °C to harvest the supernatant, and the proteins were subjected to Western blot analysis, as described previously. For the MDA assay, tissue proteins were prepared according to the manufacturer's instructions for the Lipid Peroxidation MDA assay kit (Beyotime). The MDA concentration of each sample was evaluated by multimode microplate readers (SpectraMax M5) at 532 nm using 490 nm as a control.

### Immunohistochemistry

Immunohistochemical studies were performed on paraformaldehyde-fixed, paraffin-embedded sections. Tumor samples were fixed in 4% paraformaldehyde solution and tissues were embedded in paraffin and cut at 5 μm. After deparaffinization and appropriate epitope retrieval, the sections were incubated with rabbit anti-Notch1 and rabbit anti-CD14 antibodies. The sections were further incubated with biotinylated goat anti-rabbit antibodies. The specific signals were then detected with streptavidin-conjugated horseradish peroxidase and with the use of diaminobenzidine as the chromogen.

### Statistical analysis

The results are expressed as the means ± standard deviation (SD), and all statistical analyses were performed using Student's t-test (two-tailed, unpaired). A P-value of 0.05 or less was considered significant.
